# Mindfulness, climate anxiety and psychological distress: a pathway to pro-environmental action in Pakistani emerging adults

**DOI:** 10.1017/gmh.2026.10234

**Published:** 2026-05-26

**Authors:** Nuzhat Bashir, Iram Gul, Rakhshanda Liaqat

**Affiliations:** 1 Department of Behavioural Sciences, Fatima Jinnah Women University, Rawalpindi, Pakistan; 2 Department of Psychology, International Islamic University Islamabad, Pakistan; 3 Perinatal Mental Health, HDRF, Pakistan

**Keywords:** climate anxiety, mindfulness, psychological distress, pro-environmental behavior, Pakistan

## Abstract

Climate-related worry is increasingly recognized as a mental health concern and a potential correlate of pro-environmental behavior (PEB). However, little is known about these associations in the Global South or the potential role of mindfulness. This crosssectional study examined relationships among climate change anxiety, psychological distress, mindfulness, and PEB in 340 university students (aged 19–25 years) from Islamabad and Rawalpindi, Pakistan. Data were collected between January and June 2024 using validated measures of climate change anxiety (Climate Change Anxiety Scale), psychological distress (DASS-21), dispositional mindfulness (MAAS), and PEB. Analyses included bivariate correlations, block-wise linear regression, and mediation models (PROCESS Model 4; 5,000 bootstrap resamples). Climate change anxiety was positively correlated with psychological distress (r = .47, p < .001) and indirectly associated with lower PEB through distress (ab = −.09, 95% CI [−.15, −.04]). Regression analyses showed that mindfulness was positively associated with PEB (β = .27, p < .001), whereas distress (β = −.22, p = .002) and climate change anxiety (β = −.18, p = .018) were negatively associated with PEB. Mindfulness was indirectly associated with higher PEB through lower distress (ab = .11, 95% CI [.05, .19]) and lower climate change anxiety (ab = .08, 95% CI [.02, .15]), while also showing a direct positive association with PEB. These findings highlight mindfulness as a relevant factor for understanding climate-related distress and environmental engagement among young adults.

## Impact statement

Climate change is not only an environmental crisis but also a growing mental health concern. Young people in low- and middle-income countries are especially vulnerable because they live with the daily reality of climate disasters, yet their experiences remain overlooked in research. This study helps fill that gap by focusing on emerging adults in Pakistan, one of the most climate-affected countries in the world. We found that climate anxiety is closely linked to higher levels of psychological distress. This distress can leave young people feeling paralyzed, making it harder for them to take part in the pro-environmental behaviors that are urgently needed. At the same time, our study highlights an important protective factor: mindfulness. Young people who practiced greater mindfulness not only showed more pro-environmental behavior but also experienced less climate anxiety and psychological distress. These findings matter for both public health and climate action. They show that addressing the mental health impact of climate change is not a secondary concern, but a central part of building resilience and supporting sustainable behavior. They also point to mindfulness as a practical and adaptable solution. Integrating mindfulness-based approaches into schools, communities and digital platforms could help vulnerable youth cope with the emotional weight of the climate crisis while also empowering them to take constructive, meaningful action. Ultimately, these insights extend beyond Pakistan, offering lessons for other climate-vulnerable settings worldwide.

## Introduction

Climate change is no longer a distant environmental concern but an urgent public health crisis, with consequences that extend beyond rising temperatures and extreme weather to profoundly affect mental health and well-being. A growing body of research documents a psychological response termed climate change anxiety, which reflects persistent worry, despair and fear about the planet’s future (Pihkala, 2022). Unlike clinical anxiety disorders, climate change anxiety is grounded in real environmental threats, yet its intensity can significantly impair functioning. Recognizing this, the American Psychological Association and other global health bodies have emphasized climate change as a critical psychological challenge, warning that its effects on mental health will be long-lasting and unequally distributed across populations (Thoma et al., 2021).

Young people appear especially vulnerable to these effects. Developmental psychology highlights that adolescence and early adulthood are periods of identity formation, future planning and heightened sensitivity to uncertainty (Ojala et al., 2021). Exposure to narratives of climate catastrophe during this life stage can undermine a sense of safety, agency and hope. International surveys underscore this vulnerability: in a landmark study across 10 countries, over 60% of young people reported being “very” or “extremely” worried about climate change, and nearly half stated that these worries disrupted daily life (Hickman et al., 2021). Emotional responses range from sadness and helplessness to anger at perceived government inaction, illustrating the multidimensional nature of climate change anxiety. While a degree of concern may encourage adaptive behavior and climate activism, overwhelming anxiety is associated with depression, generalized anxiety and hopelessness (Schwartz et al., 2023).

However, most empirical research on climate change anxiety comes from high-income countries in Europe, North America and Australia (Niedzwiedz et al., 2025). In these contexts, young people are exposed to global climate narratives but are often relatively shielded from the immediate devastation of floods, droughts or food insecurity. By contrast, young people in low- and middle-income countries (LMICs) face what has been termed “double exposure”: they are simultaneously immersed in catastrophic climate discourses and directly affected by local environmental disasters (Cianconi et al., 2020). Pakistan provides a striking example. Ranked among the 10 most climate-vulnerable nations (Arshed et al., 2024), the country has faced recurrent floods, droughts, heatwaves and glacial melt. The devastating 2022 floods displaced over 33 million people, destroyed essential infrastructure and disrupted education for millions of children and adolescents (Sujaya et al., 2023). Such crises not only cause physical damage but also erode young people’s sense of stability and hope. Yet, despite this acute vulnerability, the psychological experiences of youth in Pakistan remain largely absent from the literature.

Understanding not only the prevalence but also the mechanisms linking climate change anxiety to behavior is crucial. Research suggests that while some young people respond to climate-related worry with paralysis, others channel their concern into constructive pro-environmental behaviors (PEBs) (Stanley et al., 2021). Why some experience debilitating distress while others adapt remains poorly understood. Resilience theory provides a useful lens, emphasizing psychosocial resources that buffer stress and promote coping (Banerjee et al., 2021). However, the present study focuses specifically on mindfulness as a resilience resource, rather than the broader set of factors discussed in some resilience frameworks (Martin et al., 2020).

Mindfulness is emerging as a potentially important resource. Defined as non-judgmental attention to the present moment (Brown and Ryan, 2003; Kormos and Gifford, 2014), mindfulness has been associated with lower psychological distress, reduced rumination and greater emotional regulation (Pidgeon and Keye, 2014). In the environmental domain, mindful individuals may be more aware of their consumption patterns and more intentional in their behaviors, potentially linking mindfulness to pro-environmental action (Panno et al., 2018). Mindfulness may also influence how individuals experience climate-related worry. Rather than suppressing concern, mindful individuals may approach it with acceptance, allowing anxiety to coexist with constructive action rather than becoming paralyzing (Izzaturrahmah and Fauzan, 2024).

Despite these theoretical insights, few studies have examined how such psychosocial factors shape the relationship between climate anxiety, psychological distress and behavioral outcomes, particularly in LMICs. Much of the existing literature treats climate anxiety as a predictor or outcome rather than exploring the pathways through which it operates (Whitmarsh et al., 2022). In highly climate-vulnerable countries like Pakistan, where both climate shocks and community reliance are profound, testing such pathways is particularly important. Doing so not only extends theoretical understanding but also highlights potential advantage points for interventions to strengthen youth resilience and engagement in PEB (Ghorbanzadeh et al., 2025). To address these gaps, the present study examined the relationships among mindfulness, climate change anxiety, psychological distress and PEB in Pakistani university students. Specifically, we addressed three research questions:

RQ1: What are the bivariate associations among mindfulness, climate change anxiety, psychological distress and PEB?

RQ2: After accounting for demographic characteristics, are mindfulness, climate change anxiety and psychological distress independently associated with PEB?

RQ3: Are the associations between (1) climate change anxiety and PEB and (2) mindfulness and PEB, consistent with indirect pathways through psychological distress and climate change anxiety, respectively?

Based on prior research, we hypothesized that (1) climate change anxiety would be positively correlated with psychological distress; (2) mindfulness would be negatively correlated with both distress and climate change anxiety and positively correlated with PEB and (3) the patterns of associations would be consistent with indirect pathways wherein psychological distress mediates the link between climate change anxiety and PEB, and both distress and climate change anxiety mediate the link between mindfulness and PEB.

## Method

### Research design

This study employed a cross-sectional survey design to investigate the relationships among mindfulness, climate change anxiety, psychological distress and PEB in young adults. Data were gathered at a single point in time using standardized self-report questionnaires. A cross-sectional approach was considered appropriate because it enabled efficient assessment of key psychological constructs and behavioral outcomes within a large university-based sample.

### Participants and sampling

Participants were recruited from universities in the twin cities of Rawalpindi and Islamabad, Pakistan. These cities were selected as they represent major educational hubs with diverse student populations from across the country. While not directly affected by the 2022 floods, students in these cities are exposed to climate change discourse and may have family connections to affected regions. University students were specifically targeted because emerging adulthood is a critical period for identity formation, future planning and susceptibility to climate-related worry (Ojala et al., 2021). Age limits of 19–25 years were imposed to capture this developmental stage.

Institutional permissions were obtained, and a convenience sampling approach was used. Recruitment involved on-campus flyers, class announcements and online invitations shared through student networks. A total of 390 students were invited to participate, of whom 364 provided consent and completed the questionnaires. After excluding incomplete responses, the final analytic sample comprised 340 students.

- *Sample size justification.* An a priori power analysis was conducted using G*Power 3.1 for multiple linear regression with up to six predictors. Assuming a small-to-medium effect size (f^2^ = .10), α = .05 and power = .80, the required sample size was approximately 200 participants. For mediation analysis, simulation-based recommendations suggest that samples exceeding 300 are adequate for detecting small-to-medium indirect effects with bias-corrected bootstrapping (Fritz and MacKinnon, 2007). The achieved sample of N = 340 exceeds both criteria.

### Research setting

The study was carried out in Rawalpindi and Islamabad, two major educational hubs of Pakistan. Islamabad hosts prominent institutions, such as Quaid-i-Azam University, COMSATS University and the National University of Sciences and Technology, which attract a diverse student body from across the country. Rawalpindi includes Fatima Jinnah Women University and Rawalpindi Medical University, among others. Conducting the study across these urban academic centers provided access to students from varied academic disciplines and socioeconomic backgrounds, enhancing the diversity and external validity of the sample.

### Measures

- *Demographic information.* Students reported demographic details, including age, gender, family system, number of siblings, marital status, employment status, household income and recent climate-related traumatic experiences (e.g., exposure to floods, heatwaves or other climate-related disasters within the past 5 years).
- *Mindfulness.* Mindfulness was measured using the *Mindful Attention Awareness Scale* (Brown and Ryan, 2003). This 15-item instrument assesses receptive awareness of present-moment experiences. Items are rated on a six-point Likert scale from 1 (“almost always”) to 6 (“almost never”), with higher scores reflecting greater mindfulness. Total scores range from 15 to 90. The MAAS has been widely validated internationally and has been used in South Asian populations. Internal consistency in the present sample was good (Cronbach’s α = .86).
- *Climate change anxiety.* Climate-related anxiety was assessed with the *Climate Change Anxiety Scale* (Clayton and Karazsia, 2020). The 13 items measure cognitive and functional impairment due to preoccupation with climate change, such as difficulty concentrating, intrusive thoughts or sleep problems. Items are rated on a five-point Likert scale (1 = “never” to 5 = “almost always”). Total scores range from 13 to 65, with higher scores indicating greater climate anxiety. The CCAS has been validated in multiple cultural contexts. In this study, reliability was excellent (α = .90).
- *Psychological distress.* General psychological distress was assessed with the *Depression Anxiety Stress Scales 21* (Lovibond and Lovibond, 1995). The scale includes three subscales (depression, anxiety, stress), each comprising seven items. Responses are scored from 0 (“did not apply to me at all”) to 3 (“applied to me very much or most of the time”). Subscale scores are summed and doubled to determine severity levels, with each subscale ranging from 0 to 42. The DASS-21 has been validated in South Asian populations, including Pakistani samples (cite relevant validation study if available). Reliability in this sample was strong (α = .92 for depression, .90 for anxiety and .88 for stress).
- *Pro-environmental behavior.* PEB was measured with the *Pro-Environmental Behavior Scale* (Whitmarsh and O’Neill, 2010), a 24-item instrument that evaluates environmentally responsible actions such as recycling, energy conservation, sustainable transportation and mindful consumption. Items were adapted slightly for the Pakistani context (e.g., references to specific recycling programs were modified to reflect local infrastructure, and transportation items were adjusted to include locally relevant modes such as rickshaws and minibuses). A full list of adapted items is available from the corresponding author. Items are scored on a Likert scale, with total scores ranging from 24 to 120; higher scores reflect greater engagement in pro-environmental practices. Internal consistency was excellent in this sample (α = .92).

### Procedure

Data were collected between January and June 2024. Students expressing interest received an information sheet detailing the study’s objectives, procedures, potential risks and benefits. Written informed consent was obtained before participation. Surveys were completed either in person on university campuses or online through a secure platform. On average, students required 30–35 minutes to complete the full set of questionnaires. Responses were anonymous, and all data were stored in password-protected files accessible only to the research team.

### Data analysis

All analyses were conducted using SPSS version 28 with the PROCESS macro (Hayes 2022) for mediation analyses. Data screening. Data were examined for missing values, normality and outliers. Less than 2% of data were missing across all variables. Little’s Missing Completely at Random (MCAR) test indicated that data were missing completely at random (χ^2^ = 24.56, df = 28, p = .65). Missing data were handled using listwise deletion, as the proportion was minimal and MCAR assumptions were satisfied. Normality was assessed through skewness and kurtosis values, all of which fell within acceptable ranges (±2). Outliers were examined using boxplots and standardized scores; no extreme outliers requiring removal were identified.

- *Preliminary analyses.* Descriptive statistics (means, standard deviations, frequencies and percentages) were calculated for demographic and study variables. Scale reliabilities were assessed using Cronbach’s alpha.
- *Bivariate correlations.* Pearson correlations were used to examine associations among mindfulness, climate change anxiety, psychological distress and PEB. All correlation coefficients are reported with 95% confidence intervals (CIs).
- *Regression analyses.* To identify predictors of PEB, block-wise (sequential) linear regression analyses were performed, with demographic variables entered in the first block and psychological predictors added in subsequent steps. Regression assumptions (linearity, independence of errors, homoscedasticity, normality of residuals and absence of multicollinearity) were assessed and satisfied. Variance inflation factors were below 2.0 for all predictors, indicating no multicollinearity concerns. Group comparisons. Group comparisons (e.g., by gender, family system or trauma exposure) were conducted using independent-samples t-tests.
- *Mediation analyses.* Mediation analyses were performed using Hayes’s PROCESS macro (Model 4; Hayes, 2013) with 5,000 bootstrap resamples to generate bias-corrected 95% CIs for indirect effects. Two models were tested (1) whether psychological distress mediated the link between climate change anxiety and PEB and (2) whether psychological distress and climate change anxiety parallel-mediated the relationship between mindfulness and PEB. All mediation models were adjusted for age, gender, family system, household income and climate-related trauma exposure.
- *Interpretation caveat.* Due to the cross-sectional design, mediation analyses estimate statistical indirect effects consistent with, but not proof of, the hypothesized theoretical model.

### Ethical considerations

The study received ethical approval from the Institutional Review Board of Fatima Jinnah Women University, Rawalpindi (FJWU/EC/2024/74). Permissions were also obtained from the authors of the assessment instruments. Participants were informed that their involvement was voluntary, and they could withdraw at any stage without penalty. Confidentiality and anonymity were ensured throughout. Considering the sensitive nature of topics such as distress and climate anxiety, participants were provided with contact details of counseling services should the survey cause discomfort.

## Results

### Participant characteristics

A total of 340 university students participated in the study. The mean age of participants was 21.8 years (SD = 2.1, range = 19–25). Slightly more than half of the sample identified as female (54.7%, n = 186), and 45.3% (n = 154) identified as male. The majority reported living in a joint family system (61.8%, n = 210), while 38.2% (n = 130) lived in nuclear households. In terms of education, 34.4% (n = 117) had completed intermediate education, 57.6% (n = 196) were undergraduates and 8.0% (n = 27) were graduate students. Most participants were single (82.6%, n = 281), with 17.4% (n = 59) married or engaged. A quarter of the sample (25.9%, n = 88) were employed. Median monthly household income was PKR 95,000 (IQR = 70,000–150,000). Regarding family size, 10.6% (n = 36) had one sibling, 38.8% (n = 132) had two to three siblings and 50.6% (n = 172) had four or more siblings. A small proportion of participants (11.2%, n = 38) reported prior exposure to climate-related traumatic events (e.g., floods, heatwaves). Detailed sociodemographic characteristics are presented in Table 1.

**Table 1.** Sociodemographic characteristics of the sample (N = 340) Table 1. long description.

Characteristic	n	%	M (SD)/Median (IQR)
Age (years)	–	–	21.8 (2.1)
**Gender**			
Male	154	45.3	–
Female	186	54.7	–
**Education**			
Intermediate	117	34.4	–
Undergraduate	196	57.6	–
Graduate	27	8.0	–
**Marital status**			
Single	281	82.6	–
Married/engaged	59	17.4	–
**Family system**			
Joint	210	61.8	–
Nuclear	130	38.2	–
**Employment status**			
Employed	88	25.9	–
Unemployed	252	74.1	–
**Household monthly income (PKR)**	–	–	95,000 (70,000–150,000)
**Number of siblings**			
1 sibling	36	10.6	–
2–3 siblings	132	38.8	–
≥4 siblings	172	50.6	–
**Climate-related trauma exposure**			
Yes	38	11.2	–
No	302	88.8	–

*Note:* Income is reported as median and interquartile range due to non-normal distribution.

### Descriptive statistics and bivariate correlations

Descriptive statistics and Pearson correlations among the study variables are presented in Table 2. The mean mindfulness score was 54.3 (SD = 9.7), the mean climate change anxiety score was 32.5 (SD = 8.1), the mean psychological distress score was 41.2 (SD = 10.4) and the mean PEB score was 93.0 (SD = 19.7).

**Table 2.** Descriptive statistics and Pearson correlations among study variables (N = 340) Table 2. long description.

Variable	M	SD	1	2	3
1. Mindfulness	54.3	9.7			
2. Climate change anxiety	32.5	8.1	−.29***		
			[−.38, −.19]		
3. Psychological distress	41.2	10.4	−.41***	.47***	
			[−.49, −.32]	[.38, .55]	
4. Pro-environmental behavior	93.0	19.7	.32***	−.25***	−.34***
			[.22, .41]	[−.35, −.15]	[−.43, −.24]

*Note:* Values in brackets are 95% confidence intervals. ***p < .001.

Higher mindfulness scores were moderately and negatively correlated with climate change anxiety (r = −.29, 95% CI [−.38, −.19], p < .001) and psychological distress (r = −.41, 95% CI [−.49, −.32], p < .001), and positively correlated with PEB (r = .32, 95% CI [.22, .41], p < .001). Climate change anxiety showed a moderate positive correlation with psychological distress (r = .47, 95% CI [.38, .55], p < .001) and negative correlations with both mindfulness (r = −.29, 95% CI [−.38, −.19], p < .001) and PEB (r = −.25, 95% CI [−.35, −.15], p < .001). Psychological distress was also negatively correlated with PEB (r = −.34, 95% CI [−.43, −.24], p < .001).

All reported correlations were statistically significant at p < .001, with 95% CIs indicating moderate effect sizes. Given that all variables were measured simultaneously, these findings are interpreted as correlations rather than directional or causal relationships.

### Block-wise linear regression analyses

Block-wise (sequential) linear regression analyses were conducted to examine adjusted associations with PEB. Sociodemographic variables (gender, age, family system) were entered in the first block, followed by psychological variables (mindfulness, climate change anxiety, psychological distress) in the second block. Results are presented in Table 3.

**Table 3.** Block-wise linear regression examining adjusted associations with pro-environmental behavior (N = 340) Table 3. long description.

Predictor	β	SE	95% CI	t	p
Gender	.08	.05	[−.02, .18]	1.51	.132
Age	.06	.05	[−.04, .16]	1.12	.262
Family system	.11	.05	[.01, .21]	2.07	.039
Mindfulness	.27	.06	[.15, .39]	4.91	< .001
Climate change anxiety	−.18	.08	[−.34, −.02]	−2.37	.018
Psychological distress	−.22	.07	[−.36, −.08]	−3.14	.002

Model statistics: R^2^ = .28; ΔR^2^ = .21; F(6, 333) = 21.56, p < .001. *Note:* Standardized coefficients were reported. Family system coded: 1 = joint, 0 = nuclear. Because psychological distress and climate anxiety may lie on the causal pathway from mindfulness to PEB, these coefficients represent direct associations (i.e., associations independent of the other psychological variables).

In the final model, greater mindfulness was positively associated with PEB (β = .27, SE = .06, 95% CI [.15, .39], t = 4.91, p < .001). In contrast, higher climate change anxiety (β = −.18, SE = .08, 95% CI [−.34, −.02], t = −2.37, p = .018) and higher psychological distress (β = −.22, SE = .07, 95% CI [−.36, −.08], t = −3.14, p = .002) were negatively associated with PEB.

Among sociodemographic variables, living in a joint family system showed a small but statistically significant positive association with PEB (β = .11, SE = .05, 95% CI [.01, .21], t = 2.07, p = .039), while age (β = .06, SE = .05, 95% CI [−.04, .16], t = 1.12, p = .262) and gender (β = .08, SE = .05, 95% CI [−.02, .18], t = 1.51, p = .132) were not significantly associated.

The full model explained 28% of the variance in PEB (R^2^ = .28, F(6, 333) = 21.56, p < .001), with the addition of psychological variables accounting for a substantial increase in explained variance (ΔR^2^ = .21, p < .001).

All coefficients are reported as standardized estimates with corresponding standard errors and 95% CIs.

### Mediation analyses

**Figure 1.**
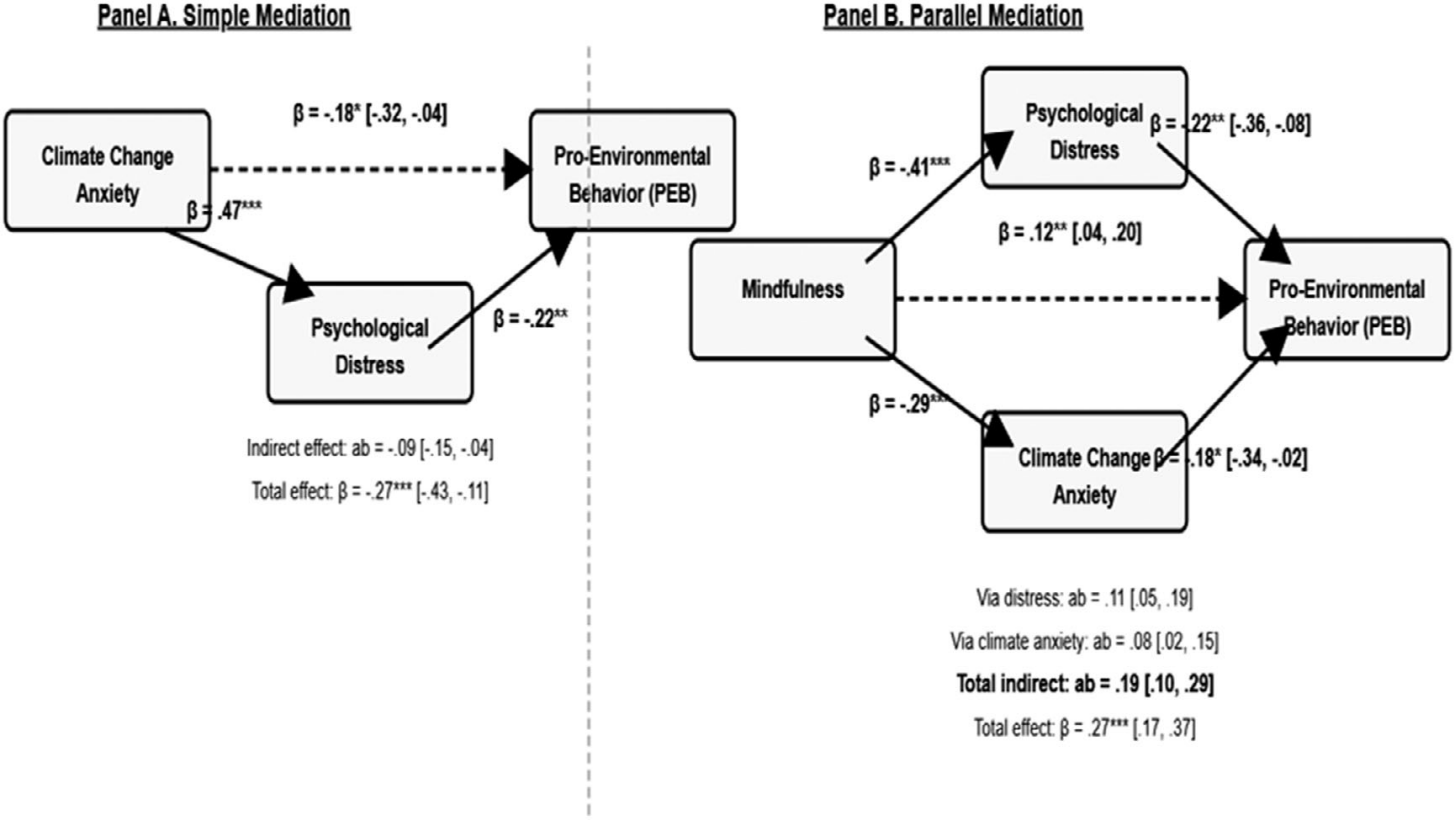
Exploratory mediation models examining indirect associations with pro-environmental behavior. *Note:* Values in brackets represent 95% bootstrap confidence intervals (5,000 resamples). All models adjusted for age, gender, family system, household income and climate-related trauma exposure. PEB = pro-environmental behavior. Figure 1. long description.

Exploratory mediation analyses were conducted to examine indirect associations among mindfulness, climate change anxiety, psychological distress and PEB. All mediation models were adjusted for age, gender, family system, household income and climate-related trauma exposure. Results are presented in Table 4 and illustrated in Figure 1.

**Table 4.** Exploratory mediation analyses examining indirect associations with pro-environmental behavior Table 4. long description.

Panel A. Simple mediation			
Climate change anxiety → Psychological distress → Pro-environmental behavior			
Effect	Coefficient	Boot SE	95% Boot CI
Total effect (climate anxiety → PEB)	−.27	.08	[−.43, −.11]
Direct effect (climate anxiety → PEB)	−.18	.07	[−.32, −.04]
Indirect effect (via distress)	−.09	.03	[−.15, −.04]

Panel B. Parallel mediation			
Mindfulness → Psychological distress/Climate change anxiety → Pro-environmental behavior			
Indirect path	Effect	SE	95% CI
Via psychological distress	.11	.04	[.05, .19]
Via climate change anxiety	.08	.03	[.02, .15]
Total indirect effect	.19	.05	[.10, .29]
Total effect (mindfulness → PEB)	.27	.05	[.17, .37]
Direct effect (mindfulness → PEB)	.12	.04	[.04, .20]

*Note:* Standardized coefficients were reported. Bootstrap confidence intervals based on 5,000. All mediation models adjusted for age, gender, family system, household income, and climate-related trauma exposure. PEB = pro-environmental behavior. Due to the cross-sectional design, these findings represent statistical indirect effects consistent with the hypothesized theoretical model, not evidence of causal mediation.

In the simple mediation model (panel A of Table 4), psychological distress partially accounted for the relationship between climate change anxiety and PEB. The total effect of climate change anxiety on PEB was significant (β = −.27, 95% Boot CI [−.43, −.11]). The indirect effect via psychological distress was statistically significant (ab = −.09, 95% Boot CI [−.15, −.04]), while the direct effect remained significant (β = −.18, 95% Boot CI [−.32, −.04]), indicating partial mediation.

In the parallel mediation model (panel B of Table 4), the association between mindfulness and PEB was indirectly explained through both lower psychological distress and lower climate change anxiety. The indirect effect via psychological distress was significant (ab = .11, 95% CI [.05, .19]), and the indirect effect via climate change anxiety was also significant (ab = .08, 95% CI [.02, .15]). The total indirect effect was .19 (95% CI [.10, .29]), accounting for a substantial proportion of the total association. The total effect of mindfulness on PEB was significant (β = .27, 95% CI [.17, .37], p < .001), and the direct effect remained significant after accounting for both mediators (β = .12, 95% CI [.04, .20], p = .004), indicating partial mediation.

All mediation estimates are reported as standardized coefficients with bootstrap-based 95% CIs based on 5,000 resamples. Due to the cross-sectional design, these findings are interpreted as statistical indirect effects consistent with the hypothesized theoretical model, rather than evidence of causal mediation.

## Discussion

This study examined associations among mindfulness, climate change anxiety, psychological distress and PEB in a sample of Pakistani university students. The findings revealed that mindfulness was positively associated with PEB, while climate change anxiety and psychological distress were negatively associated with PEB at both the bivariate and multivariable levels. Mediation analyses indicated that the associations were consistent with indirect pathways: climate change anxiety was linked to lower PEB partly through higher psychological distress, and mindfulness was linked to higher PEB partly through lower psychological distress and lower climate change anxiety. These patterns align with the hypothesized theoretical model, though the cross-sectional design precludes causal inferences.

The mediation model tested in this study represents one theoretically plausible configuration among several alternatives. Given the cross-sectional design, alternative temporal orderings cannot be ruled out. For example, psychological distress may precede or shape climate-related anxiety or engagement in PEB may influence psychological states through increased perceived agency or meaning. These alternative interpretations highlight the need for longitudinal and experimental designs to clarify directionality and causal pathways.

The finding that climate change anxiety was positively correlated with psychological distress (r = .47) is consistent with prior research documenting the mental health correlates of climate-related worry. Hickman et al. (2021) reported that over 60% of youth across 10 countries experienced climate anxiety that interfered with daily functioning, and Clayton and Karazsia (2020) found that climate change anxiety was associated with poorer mental health outcomes in U.S. samples. The present study extends these findings to a climate-vulnerable LMIC context, where young people face both global climate narratives and local environmental disasters (Cianconi et al., 2020).

The negative association between climate change anxiety and PEB (r = −.25) contributes to a mixed literature. Some studies have found that climate concern can motivate pro-environmental action (Stanley et al., 2021), while others report that overwhelming anxiety may lead to paralysis and disengagement (Schwartz et al., 2023). Higher climate anxiety was associated with lower PEB, and this association was partially accounted for by psychological distress. This may reflect that the Climate Change Anxiety Scale primarily captures functional impairment related to climate-related preoccupation, rather than broader motivational or concern-based aspects of climate anxiety. As such, higher scores may indicate forms of anxiety that are more likely to interfere with, rather than facilitate, engagement in PEB.

The positive association between mindfulness and PEB (β = .27) aligns with research in Western contexts showing that mindful individuals report stronger environmental values and sustainable behaviors (Panno et al., 2018; Geiger et al., 2019). The present study extends this literature by demonstrating that mindfulness is associated with PEB even after accounting for distress and climate anxiety, and that these psychological factors may represent pathways through which mindfulness relates to environmental action.

The mediation findings warrant cautious interpretation given the cross-sectional design. The pattern of associations is consistent with the possibility that psychological distress serves as an intervening mechanism linking climate anxiety to lower PEB. This may occur if climate-related worry contributes to symptoms of depression, anxiety or stress that deplete the emotional and cognitive resources needed for sustained pro-environmental engagement. Alternatively, individuals experiencing higher distress may have fewer opportunities or less motivation to engage in PEB.

Similarly, mindfulness was associated with lower distress and lower climate anxiety, and these factors in turn were associated with higher PEB. This pattern is consistent with theoretical accounts suggesting that mindfulness supports emotional regulation and non-reactive awareness of distressing thoughts (Panno et al., 2018), potentially preventing climate concern from becoming functionally impairing. Mindful individuals may be better able to hold climate-related worries without becoming overwhelmed, allowing concern to coexist with constructive action.

However, alternative temporal orderings are equally plausible with cross-sectional data. For example, engaging in PEBs might reduce distress by fostering a sense of agency and purpose, or individuals with lower baseline distress may be more disposed to both mindfulness and PEB. Longitudinal and experimental designs are needed to clarify directionality.

### Limitations

This study has several limitations that should be considered when interpreting the findings.

First, the cross-sectional design precludes conclusions about causality or temporal ordering. Although the mediation analyses estimate statistical indirect effects consistent with the hypothesized model, alternative directional pathways are plausible and reverse causation cannot be ruled out.

Second, all variables were assessed using self-report measures at a single time point, which may introduce social desirability bias, particularly in reporting PEBs, and may also result in shared method variance that inflates observed associations.

Third, the sample was limited to urban, educated university students in Islamabad and Rawalpindi. These findings may not generalize to rural populations or to communities more directly exposed to climate-related disasters, where the nature, intensity and functional impact of climate-related anxiety may differ substantially.

Fourth, the Climate Change Anxiety Scale primarily captures functional impairment related to climate-related preoccupation and does not encompass the broader spectrum of climate-related emotional responses such as climate grief, anger or ecological distress. Therefore, the findings should be interpreted specifically in relation to climate-related functional impairment rather than the full construct of climate anxiety.

Fifth, psychological distress was analyzed as a composite construct including depression, anxiety and stress. Examining these subcomponents separately may reveal more nuanced relationships with climate anxiety and PEB.

Finally, potential confounding variables such as personality traits, prior mental health history, environmental attitudes and exposure to climate-related information were not assessed and may have influenced the observed relationships.

### Implications and future directions

- *Research implications.* The findings highlight several directions for future research. Longitudinal studies tracking mindfulness, climate anxiety, distress and PEB over time are needed to establish temporal precedence and examine bidirectional relationships. Experimental designs testing mindfulness-based interventions could determine whether changes in mindfulness precede changes in distress and PEB. Cross-cultural comparisons would be valuable to examine whether the observed patterns hold across diverse sociopolitical and ecological contexts.
- *In the Pakistani context specifically*, future research should investigate whether findings replicate in rural and flood-affected populations, who may experience climate anxiety differently. Studies examining culturally specific resilience factors such as religious coping, community support or indigenous ecological knowledge could identify additional protective mechanisms. Qualitative research exploring how young Pakistanis experience and make meaning of climate-related distress would enrich quantitative findings.
- *Practical implications.* If supported by longitudinal and experimental research, the findings suggest that addressing psychological distress may be important not only for mental health but also for sustaining pro-environmental engagement. Interventions that reduce distress, whether through mindfulness, cognitive-behavioral approaches or social support, could potentially remove barriers to climate action.

Mindfulness-based approaches warrant further investigation as they are relatively accessible and have been successfully adapted across cultures (González-Palau and Medrano, 2022). However, several caveats are important. First, this study did not test an intervention; the observed associations do not demonstrate that mindfulness training would effectively reduce distress or increase PEB. Second, feasibility, acceptability, cultural appropriateness and cost-effectiveness of mindfulness interventions in Pakistani educational and community settings would need to be established through rigorous evaluation. Third, mindfulness should not be positioned as a standalone solution to climate anxiety. Individual-level coping strategies must complement not replace policy-level interventions, structural changes and collective action that address the root causes of climate change (e.g., fossil fuel dependence, inadequate climate policy).

Educators and policymakers could consider integrating psychoeducation about climate-related distress into university curricula, normalizing these experiences while providing coping resources. Peer support groups and campus-based climate action initiatives may help transform anxiety into constructive engagement.

## Conclusion

Among Pakistani university students, climate change anxiety was associated with higher psychological distress, which in turn was associated with lower PEB. Mindfulness was associated with lower distress and lower climate anxiety, and these factors partially accounted for its positive association with PEB. These findings underscore the importance of attending to the psychological dimensions of the climate crisis and suggest that interventions supporting emotional regulation and distress reduction may help sustain environmental engagement. However, causal conclusions await longitudinal and experimental research, and individual-level approaches must be coupled with structural solutions. Understanding and supporting young people’s psychological responses to climate change remains a critical priority for research, policy and practice in Pakistan and beyond.

## Data Availability

The datasets generated and analyzed during this study are not publicly available due to confidentiality agreements with participants. However, de-identified data may be shared upon reasonable request to the corresponding author.

## Long descriptions

### Table 1. Long description

The table has four columns labeled Characteristic, n, percent, and M (S D) or Median (I Q R). Age is listed first with a mean of 21.8 years and standard deviation 2.1. Gender is divided into male, 154 participants (45.3 percent), and female, 186 participants (54.7 percent). Education is categorized as intermediate, 117 (34.4 percent), undergraduate, 196 (57.6 percent), and graduate, 27 (8.0 percent). Marital status includes single, 281 (82.6 percent), and married or engaged, 59 (17.4 percent). Family system is joint, 210 (61.8 percent), and nuclear, 130 (38.2 percent). Employment status is employed, 88 (25.9 percent), and unemployed, 252 (74.1 percent). Household monthly income is reported as median 95,000 P K R with interquartile range 70,000 to 150,000. Number of siblings is 1 sibling, 36 (10.6 percent), 2 to 3 siblings, 132 (38.8 percent), and 4 or more siblings, 172 (50.6 percent). Climate-related trauma exposure is yes, 38 (11.2 percent), and no, 302 (88.8 percent). Income is noted as median and interquartile range due to non-normal distribution.

Navigate back to Table 1..

### Table 2. Long description

From the top row downward, the table lists four variables: mindfulness, climate change anxiety, psychological distress, and pro-environmental behavior. For each, the mean and standard deviation are provided: mindfulness 54.3 (9.7), climate change anxiety 32.5 (8.1), psychological distress 41.2 (10.4), pro-environmental behavior 93.0 (19.7). Pearson correlations are shown in the lower triangle. Mindfulness is negatively correlated with climate change anxiety (minus point two nine, 95 percent confidence interval minus point three eight to minus point one nine, p less than point zero zero one), and with psychological distress (minus point four one, confidence interval minus point four nine to minus point three two, p less than point zero zero one). Mindfulness is positively correlated with pro-environmental behavior (point three two, confidence interval point two two to point four one, p less than point zero zero one). Climate change anxiety is positively correlated with psychological distress (point four seven, confidence interval point three eight to point five five, p less than point zero zero one) and negatively with pro-environmental behavior (minus point two five, confidence interval minus point three five to minus point one five, p less than point zero zero one). Psychological distress is negatively correlated with pro-environmental behavior (minus point three four, confidence interval minus point four three to minus point two four, p less than point zero zero one). Values in brackets are 95 percent confidence intervals. Triple asterisks indicate p less than point zero zero one.

Navigate back to Table 2..

### Table 3. Long description

The table presents block-wise linear regression results for pro-environmental behavior with 340 participants. Columns are Predictor, beta, S E, 95 percent confidence interval, t, and p. From top to bottom: Gender beta point zero eight, S E point zero five, confidence interval negative point zero two to point one eight, t one point five one, p point one three two. Age beta point zero six, S E point zero five, confidence interval negative point zero four to point one six, t one point one two, p point two six two. Family system beta point one one, S E point zero five, confidence interval point zero one to point two one, t two point zero seven, p point zero three nine. Mindfulness beta point two seven, S E point zero six, confidence interval point one five to point three nine, t four point nine one, p less than point zero zero one. Climate change anxiety beta negative point one eight, S E point zero eight, confidence interval negative point three four to negative point zero two, t negative two point three seven, p point zero one eight. Psychological distress beta negative point two two, S E point zero seven, confidence interval negative point three six to negative point zero eight, t negative three point one four, p point zero zero two. Model statistics: R squared point two eight, delta R squared point two one, F open parenthesis six, three hundred thirty-three close parenthesis equals twenty-one point five six, p less than point zero zero one. Family system coded one equals joint, zero equals nuclear. Coefficients represent direct associations, independent of other psychological variables.

Navigate back to Table 3..

### Table 4. Long description

Panel A, titled Simple mediation, examines the pathway from climate change anxiety to psychological distress to pro-environmental behavior. The first header row lists Effect, Coefficient, Boot S E, and 95 percent Boot C I. The total effect of climate anxiety on pro-environmental behavior is negative point two seven, Boot S E point zero eight, with a confidence interval from negative point four three to negative point one one. The direct effect is negative point one eight, Boot S E point zero seven, confidence interval negative point three two to negative point zero four. The indirect effect via distress is negative point zero nine, Boot S E point zero three, confidence interval negative point one five to negative point zero four. Panel B, titled Parallel mediation, examines mindfulness to psychological distress or climate change anxiety to pro-environmental behavior. The header row lists Indirect path, Effect, S E, and 95 percent C I. The indirect effect via psychological distress is point one one, S E point zero four, confidence interval point zero five to point one nine. The indirect effect via climate change anxiety is point zero eight, S E point zero three, confidence interval point zero two to point one five. The total indirect effect is point one nine, S E point zero five, confidence interval point one zero to point two nine. The total effect of mindfulness on pro-environmental behavior is point two seven, S E point zero five, confidence interval point one seven to point three seven. The direct effect is point one two, S E point zero four, confidence interval point zero four to point two zero. All coefficients are standardized. Models are adjusted for age, gender, family system, household income, and climate-related trauma exposure. P E B stands for pro-environmental behavior. The results reflect statistical indirect effects, not causal mediation.

Navigate back to Table 4..

### Figure 1. Long description

Panel A on the left displays a simple mediation model. At the left, ‘Climate Change Anxiety’ connects to ‘Psychological Distress’ below via a solid arrow labeled beta equals point four seven triple asterisk. Both boxes point to ‘Pro-Environmental Behavior’ at the right, with ‘Climate Change Anxiety’ using a dashed arrow labeled beta equals minus point one eight open bracket minus point three two comma minus point zero four close bracket, and ‘Psychological Distress’ using a solid arrow labeled beta equals minus point two two double asterisk. Below, indirect effect ab equals minus point zero nine open bracket point one five comma minus point zero four close bracket and total effect beta equals minus point two seven triple asterisk open bracket minus point four three comma minus point one one close bracket are listed. Panel B on the right shows a parallel mediation model. ‘Mindfulness’ at the left connects upward to ‘Psychological Distress’ via a solid arrow labeled beta equals point four one triple asterisk, and downward to ‘Climate Change Anxiety’ via a solid arrow labeled beta equals minus point two nine triple asterisk. Both ‘Psychological Distress’ and ‘Climate Change Anxiety’ point rightward to ‘Pro-Environmental Behavior’ with solid arrows labeled beta equals point two two double asterisk open bracket minus point three six comma minus point zero eight close bracket and beta equals minus point one eight open bracket minus point three four comma minus point zero two close bracket, respectively. ‘Mindfulness’ also connects directly to ‘Pro-Environmental Behavior’ with a dashed arrow labeled beta equals point one two double asterisk open bracket point zero four comma point two zero close bracket. Below, mediation effects are listed: via distress ab equals point one one open bracket point zero five comma point one nine close bracket, via climate anxiety ab equals point zero eight open bracket point zero two comma point one five close bracket, total indirect ab equals point one nine open bracket point one zero comma point two nine close bracket, and total effect beta equals point two seven triple asterisk open bracket point one seven comma point three seven close bracket.

Navigate back to Figure 1..
