# Supplementary material for: Mindfulness, climate anxiety and psychological distress: a pathway to pro-environmental action in Pakistani emerging adults
Source: Glob Ment Health (Camb). 2026 May 26;13:e125. doi: 10.1017/gmh.2026.10234 (PMC13312362; doi:10.1017/gmh.2026.10234)
Supplement: Peer review history

# Reviewer report: Review: Mindfulness, climate anxiety and psychological distress: a pathway to pro-environmental action in Pakistani emerging adults — R0/PR2

**DOI:** 10.1017/gmh.2026.10234.pr2

Dear Editor:

Thank you for the opportunity to review this manuscript examining associations among mindfulness, climate change anxiety, psychological distress, and pro-environmental behavior among university students in Pakistan. The topic is timely, relevant, and important within the global mental health and climate change literature, particularly for LMIC contexts. The manuscript is clearly written and uses validated psychological scales.

However, substantial revisions are needed before this manuscript is suitable for publication. The concerns fall into several domains: conceptual framing, methodological rigor, analytical approach, reporting clarity, interpretation, and scholarly referencing. Many issues reflect overextension beyond the data and a lack of theoretical coherence.

Major Comments

1. Conceptual and Terminological Inconsistency

The manuscript uses the terms eco-anxiety, climate anxiety, climate worry, and climate change anxiety interchangeably. These constructs differ theoretically and empirically. Since the study used the Climate Change Anxiety Scale, which measures functional impairment, I encourage the authors to consistently use that terminology and avoid implying that broader eco-emotions were assessed.

The introduction and discussion should also avoid conflating unmeasured constructs (eco-anger, grief, functional eco-anxiety). This issue also appears in the abstract, where “eco-anxiety” is used despite not being measured.

2. Weak, Unfocused, and Overextended Theoretical Framework

The introduction is long and occasionally padded, referencing many tangential concepts (collectivism, resilience, identity development, social support, nature connectedness) that do not appear in the conceptual model or results. The selection of mindfulness, climate anxiety, distress, and PEB appears driven by available survey items rather than a theory-driven rationale.

More importantly, the research questions implied in the introduction do not align with the analyses conducted. For example:

• The introduction suggests an exploration of “factors shaping climate anxiety,” yet no model uses climate anxiety as an outcome.

• Climate anxiety is discussed as a mechanism, but is not tested as a mediator or moderator.

• The theoretical framing implies multiple pathways, but the analysis only tests a single mediator (distress) for mindfulness → PEB.

The study would benefit from a clearer, more focused conceptual framework and an analytic plan tied explicitly to that framework.

3. Overinterpretation of Cross-Sectional Mediation

The manuscript frequently uses causal or mechanistic language (“mechanisms,” “pathways,” “reduces,” “fosters,” “buffers,” “explains,” “transforms”). Cross-sectional mediation cannot establish temporal precedence or causal processes.

Results should be described as associations consistent with mediation, not mechanisms. All causal language should be revised.

4. Lack of Covariate Adjustment

Although demographic variables were collected and included in Step 1 of the hierarchical regression, they were not included in the mediation analyses. This omission raises substantial concerns about omitted-variable bias. Gender, age, socioeconomic status, and climate trauma exposure are all theoretically linked to mindfulness, distress, climate anxiety, and PEB, and should be included as covariates or justified if omitted.

Additionally, demographic information is reported in both the Methods and Results sections, which creates redundancy. It is more conventional to present these data once in the Results.

5. No Sample Size Justification

The manuscript does not discuss why N = 340 is an appropriate sample size for mediation analysis, nor does it cite power analyses or relevant methodological recommendations. Including a justification (e.g., simulation-based recommendations or prior CCAS/MAAS studies with similar sample sizes) would improve analytic transparency.

6. Tables and Figures Are Incomplete and Inconsistent

There are substantial reporting issues:

• Table 1: categorical variables (e.g., married/engaged <10%) should be collapsed for interpretability.

• Table 2: column headers (“1 2 3”) are unlabeled, and variable 4 is omitted.

• Table 3: missing SEs and 95% CIs; incomplete model statistics.

• Table 4: claimed standardized coefficients do not match figures.

• Figures 1–2: path coefficients do not correspond to Table 4; unclear whether standardized or unstandardized.

All tables and figures require major revision to meet publication standards.

7. Overgeneralization Beyond the Sample

The sample consists of university students from Islamabad and Rawalpindi, yet the manuscript repeatedly generalizes to “youth in Pakistan,” “young adults in Pakistan,” or “LMIC youth.” These generalizations are not justified. Findings should be contextualized as applying specifically to urban, educated university students.

8. Unsupported Claims About Mindfulness Interventions

The discussion and policy implications claim that mindfulness-based approaches are “feasible,” “low-cost,” “scalable,” and “suitable for youth.” The study does not examine intervention feasibility, acceptability, cultural relevance, or implementation capacity. These claims are speculative and should be removed or supported by references.

Similarly, “collective climate action” is mentioned in the discussion despite not being measured; this could be revised for clearer alignment with the actual data.

9. Discussion Section Lacks Coherence

Several paragraphs in the Discussion read like additional introduction or extend into policy advocacy. Paragraphs introduce unmeasured constructs (functional eco-anxiety, collective action, resilience pathways) and claim alignment with literature that does not correspond to the constructs measured. Additionally, certain paragraphs move toward promoting mindfulness or environmental engagement rather than neutrally interpreting data. Shifting back to a scientific tone rooted in the study’s findings would strengthen this section.

The Discussion could be strengthened by:

1. neutrally summarizing findings;

2. comparing with relevant literature;

3. interpreting results cautiously;

4. acknowledging limitations clearly;

5. identifying Pakistan-specific future directions.

10. Insufficient Scholarly Referencing

Throughout the manuscript, several key claims lack citations:

• rising eco-anxiety in LMICs;

• mindfulness reducing distress;

• scale validity (“widely validated”);

• policy or practice recommendations;

• feasibility of mindfulness interventions.

Some citations are extremely general, referring vaguely to “the eco-anxiety literature” without specific sources.

Additionally:

• MAAS, DASS-21, CCAS, and Whitmarsh PEB scales are not cited at all.

• No cross-cultural validation studies are referenced.

• The PEB scale is self-report, but potential social desirability bias is not acknowledged.

Minor Comments

1. Measurement transparency: The manuscript states that PEB items were “adapted,” but does not specify how or which items were modified.

2. Measurement details: Internal consistency values are reported inconsistently; no justification is given for summing CCAS subscales.

3. Missing data: No report of missingness or handling strategy.

4. Abstract: Uses causal and generalized language and refers to eco-anxiety, which was not measured.

5. Future directions: Reasonable but very general; not tied directly to findings or to the Pakistani context.

Conclusion

This manuscript addresses an important topic with potential to contribute to climate and mental health research in LMICs. However, substantial revisions are required to address conceptual inconsistencies, methodological limitations, causal overinterpretation, and reporting issues. With a clearer theoretical focus, more careful interpretation of cross-sectional data, improved reporting of results, and stronger alignment between claims and evidence, the manuscript would be significantly strengthened.

I hope these comments are helpful as the authors revise and refine their work.

# Reviewer report: Review: Mindfulness, climate anxiety and psychological distress: a pathway to pro-environmental action in Pakistani emerging adults — R0/PR3

**DOI:** 10.1017/gmh.2026.10234.pr3

**Authors:** James Stannah

**Affiliations:** Factor-Inwentash Faculty of Social Work, University of Toronto, Canada

Review: Mindfulness, climate anxiety, and psychological distress: A pathway to pro-environmental action in Pakistani emerging adults

Thank you for the opportunity to review this manuscript. This study examines associations between mindfulness, climate change anxiety, psychological distress, and pro-environmental behavior (PEB) among Pakistani university students in two cities, using bivariate correlations, regression models, and mediation analyses. Overall, I found the paper was well written, the topic is timely, and the findings are interesting and of potential relevance to global mental health and climate psychology, particularly given the limited empirical work on eco-anxiety from settings such as Pakistan. I also commend the authors on their efforts to integrate multiple constructs and explore possible mechanisms linking them.

I am more confident in quantitative methods, so my main comments focus on the analysis and interpretation of the findings. However, I also provide additional feedback on other aspects of the paper. My main concern is that interpretation of the results, particularly regarding directionality of associations, mediation, and causal pathways, sometimes goes beyond what can be supported by the cross-sectional design and analytic approach.

Main issues:

1) Causal language and interpretation

While the authors acknowledge in their limitations that the cross-sectional design precludes causal inference, causal and mechanistic language is used throughout the paper, particularly in the Discussion. Given the cross-sectional design and use of self-reported information, the findings should be interpreted as correlations and associations, with causal claims avoided altogether.

For example, statements such as (page 12 line 288) “mindfulness reduced psychological distress, likely by enhancing emotional regulation and reducing maladaptive rumination in the face of climate-related information” are quite strong and not supported by the study design. Directionality can’t be inferred from the cross-sectional design, so we can’t know that mindfulness reduced distress, and the mechanisms proposed in that sentence weren’t measured or tested in the study. Similar concerns apply to statements such as:

- “mindfulness served as a resilience factor that buffered these effects and promoted constructive action” (page 12, line 283)

- “mindfulness appeared to transform climate anxiety into a more function form of concern” (page 12, line 292)

- “our results highlight mindfulness as a feasible, low-cost intervention strategy” (page 13, line 314)

I recommend softening causal language throughout the manuscript to reflect associations (e.g., “was associated with”, “correlated with”, etc.), and avoiding intervention-related conclusions that weren’t explored in the study.

2) Alignment between conceptual model and regression adjustment

Block-wise regression models were used to estimate predictors of PEB, adjusting first for demographics and then for psychological variables. In the final model, coefficients are interpreted as independent predictors of PEB. However, in subsequent mediation analyses, psychological distress and climate change anxiety are conceptualised as mediators of the relationship between mindfulness and PEB.

This creates some possible confusion conceptually and in interpretation. If distress and/or climate anxiety are on the causal pathway from mindfulness, then adjusting for them in the regression models estimates a controlled direct association/effect, not a total association/effect. See this Wikipedia article: https://en.wikipedia.org/wiki/Table_2_fallacy and this paper: https://pubmed.ncbi.nlm.nih.gov/23371353/ As a result, coefficients from the fully adjusted regression model cannot necessarily be interpreted as independent or overall predictors of PEB. For example, if psychological distress mediates the association between climate anxiety and PEB, then including distress in the regression model blocks part of that association.

This isn’t necessarily a problem, if accounted for in the interpretation, but I think this section would benefit from clearer alignment between the conceptual model used to inform the pathways and the analytic strategy used to estimate predictors of PEB.

I also recommend explicitly stating whether regression coefficients (e.g., in Table 3) are intended to represent total associations or direct associations and accounting for this in the interpretation.

3) Mediation analyses and cross-sectional limitations

The mediation analyses assume a specific temporal ordering between mindfulness-->climate anxiety-->psychological distress-->PEB. However, with cross-sectional data, this ordering can’t be empirically established. While mediation analyses can estimate indirect statistical associations, estimates should not be interpreted as evidence of causal mechanisms.

I recommend reframing mediation analysis findings as exploratory associations consistent with the hypothesised theoretical model, rather than as evidence supporting that model.

Additionally, it is not clear whether key demographic covariates were included in the mediation models. If not, there is a risk of residual confounding of the estimated mediated associations. Can the authors clarify?

Additional comments and suggestions

Introduction

I felt that the introduction set up the paper well, illustrating the known links between eco-anxiety and mental health, and highlighting the lack of research on eco-anxiety from low/middle income settings such as Pakistan. This paper clearly fills an important gap in addressing this and provides novel findings in its focus on mechanisms.

However, the role of mindfulness is introduced relatively late in the introduction and with limited theoretical grounding, despite appearing to be a primary exposure in the analyses. To strengthen the empirical framing, it would be helpful to explain more how mindfulness is conceptualised and what is known about its relationship with mental health and pro-environmental behaviour.

It would also be helpful to provide an example of two of the types of behaviours included under “pro-environmental behaviour” for those less familiar.

Methods

Structure of the methods section

• The section “Participant characteristics” appears in the both the Methods and Results, and the information is duplicated. As these are technically results of the survey, rather than methods, I suggest removing this section from the Methods altogether, and keeping it in the results only.

• The Methods section could be more clearly organised as: Research Design, Participants & Sampling, Research Setting, Procedures, Measures, then Data (or Statistical) Analysis.

• Minor point: in Measures, can you add the range of possible scores for each continuous scale? That would aid interpretation.

Regression modelling

• Please clarify if the regression models are linear regressions and whether model assumptions were assessed.

• The term “hierarchical multiple regression” is potentially confusing, as it may be interpreted as multilevel modelling. Perhaps it would be clearer to say something like “block-wise (sequential) regression” or similar.

Sample and generalisability

It is good that a range of recruitment strategies were used to recruit a more diverse sample of students. However, further justification for focusing specifically on university students would be helpful. Were age limits imposed? Additionally, more context on why the two cities were selected, especially related to their relationships with climate change, would help strengthen the background and rationale.

Bivariate correlations

The use of Pearson correlations is fine as descriptive analysis, but should be interpreted cautiously given variables were measured simultaneously. I suggest referring to these estimates consistently as “correlations”. At the moment, the authors switch between “correlation” and “association”, which can be confusing when estimates are presented alongside with coefficients of the regression and mediation models. Similarly, soften the causal language when interpreting these correlations.

Results reporting

• Confidence intervals are not reported for several key estimates, including correlation and regression coefficients in Tables 2 and 3. It would be helpful to include these for interpretability and transparency.

Discussion

• In the analysis, psychological distress is treated as a composite construct, but depression, anxiety, and stress are conceptually distinct. Is this a limitation? Did the authors conduct any analyses to explore associations with the subcomponents?

• The discussion of mindfulness interventions on page 13 could be expanded. In particular, acknowledging debate around the limits of mindfulness-based approaches, and the importance of addressing structural drivers of climate anxiety, could provide a more balanced conclusion. While mindfulness may help individuals cope with distress, it doesn’t directly address the underlying social, political, and environmental conditions that create eco-anxiety. Explicitly recognising this distinction could help avoid over positioning mindfulness as a standalone intervention, placing it more along wider social and policy-level responses.

In summary, I found this to be a well and clearly written manuscript, with interesting and potentially important findings. Addressing the issues above, particularly around causal language and the analytic approach, would help strengthen the paper and ensure the conclusions are well aligned with the data and methods used.

# Editor report: Recommendation: Mindfulness, climate anxiety and psychological distress: a pathway to pro-environmental action in Pakistani emerging adults — R0/PR4

**DOI:** 10.1017/gmh.2026.10234.pr4

**Affiliations:** University of Toronto Factor-Inwentash Faculty of Social Work, Canada

As you can see, both reviewers noted substantial limitations to the current manuscript with extensive suggested revisions. They also thought the topic was important and timely. The authors are invited to do this major revision, focusing on addressing each point in the reviews, which include reworking the background, methods, analysis and write up of results and discussion.

# Editor report: Decision: Mindfulness, climate anxiety and psychological distress: a pathway to pro-environmental action in Pakistani emerging adults — R0/PR5

**DOI:** 10.1017/gmh.2026.10234.pr5

**Affiliations:** London School of Hygiene & Tropical Medicine, United Kingdom of Great Britain and Northern Ireland

No accompanying comment.

# Reviewer report: Review: Mindfulness, climate anxiety and psychological distress: a pathway to pro-environmental action in Pakistani emerging adults — R1/PR7

**DOI:** 10.1017/gmh.2026.10234.pr7

Overall, the manuscript has been substantially strengthened in response to prior feedback. The revisions meaningfully improve conceptual clarity, analytic rigor, reporting transparency, and interpretive caution. The remaining suggestions are minor and primarily concern phrasing, organization, and theoretical refinement. With these addressed, the manuscript would make a valuable contribution to the climate and global mental health literature. I would be comfortable with the editor assessing the revised manuscript without an additional round of external review.

Recommendation: Minor Revision

Conceptual/Theoretical Framework

The revised manuscript presents a clearer and more focused conceptual framework than the previous version. The alignment between the research questions and the analytic models is now substantially improved, and the removal of tangential constructs strengthens coherence.

One remaining point concerns the directional ordering of variables in the mediation model. The pathway tested (mindfulness → climate change anxiety → psychological distress → pro-environmental behavior) is theoretically plausible and logically structured. However, as with most cross-sectional mediation models, alternative orderings are also conceivable. For example, climate anxiety could plausibly function as an outcome of distress, or pro-environmental behavior could relate bidirectionally with psychological states.

This does not undermine the current analysis, but theoretical transparency would be strengthened if the Discussion briefly acknowledged that the tested model represents one plausible configuration among several alternatives, and that longitudinal or experimental designs would be required to clarify temporal ordering.

Additionally, a brief clarification of the conceptual role of climate change anxiety in the model — whether primarily as functional impairment, motivational concern, or both — would further enhance theoretical precision.

These refinements are minor but would strengthen the theoretical grounding of the study.

Methods Section

The Methods section has improved substantially in clarity and transparency. One minor stylistic suggestion concerns the acknowledgment of potential social desirability bias in self-reported pro-environmental behavior (Kormos and Gifford 2014).

While the acknowledgment is appropriate, it may fit more naturally in the Discussion as part of the limitations rather than within the Methods. The Methods could remain focused on measurement description, with interpretive considerations reserved for the limitations subsection.

Results Section

The Results section is substantially improved and now reports the regression and mediation analyses clearly, with appropriate inclusion of covariates, standardized coefficients, and bootstrap confidence intervals. Analytic transparency is much stronger than in the previous version.

The remaining suggestions relate primarily to organization and streamlining. In a few places, the Results include interpretive or design-related clarifications (e.g., reminders about cross-sectional limitations or explanations of analytic structure) that may fit more naturally in the Methods or Discussion. Consolidating these caveats in one location would help maintain a clearer separation between reporting and interpretation.

Additionally, portions of the Results — particularly the “Summary of Findings” subsection — repeat numerical results already presented. This section could be shortened or removed to avoid redundancy and to focus on synthesizing overall patterns rather than restating coefficients.

Finally, the brief re-description of the block-wise regression procedure in the Results appears somewhat repetitive of the Methods and could be streamlined for concision.

Overall, the Results section is methodologically sound; the suggestions above are intended to improve clarity and flow rather than to indicate substantive concerns.

Discussion Section

The Discussion is substantially improved and now more closely aligned with the study’s design and findings. The authors appropriately frame the results as associative and acknowledge the limitations of cross-sectional mediation. Interpretation is generally cautious, and the contextualization within a sample of Pakistani university students is clearer than in the previous version.

A few minor refinements could further strengthen the section. In some places, phrasing could be tightened slightly to avoid implying directional processes beyond what cross-sectional data can support. Additionally, where alternative temporal orderings are proposed, briefly citing relevant literature or softening the language to indicate theoretical plausibility rather than established mechanisms would enhance scholarly grounding.

Ensuring that interpretations of pro-environmental behavior remain anchored to self-reported tendencies rather than broader behavioral impact would also improve precision.

Overall, the Discussion is coherent and balanced, and the suggestions above are refinements rather than indications of substantive concerns.

Limitations

The Limitations section appropriately acknowledges the cross-sectional design, reliance on self-report measures, and the restricted sampling frame. One minor suggestion would be to briefly note the potential for shared method variance given that all constructs were measured via self-report at a single time point. Additionally, acknowledging possible differences between urban university students and more climate-exposed populations within Pakistan could further contextualize generalizability. These are minor refinements rather than substantive concerns.

# Reviewer report: Review: Mindfulness, climate anxiety and psychological distress: a pathway to pro-environmental action in Pakistani emerging adults — R1/PR8

**DOI:** 10.1017/gmh.2026.10234.pr8

**Authors:** James Stannah

**Affiliations:** Factor-Inwentash Faculty of Social Work, University of Toronto, Canada

I have reviewed the manuscript and the authors' responses to my comments. I’m impressed with the edits made, which substantially improve the manuscript. I am satisfied that the authors have addressed all my concerns, and those of the other reviewer, and I have no further comments. I therefore recommend the manuscript for publication.

Many thanks for the opportunity to review this interesting paper.

# Editor report: Recommendation: Mindfulness, climate anxiety and psychological distress: a pathway to pro-environmental action in Pakistani emerging adults — R1/PR9

**DOI:** 10.1017/gmh.2026.10234.pr9

**Affiliations:** University of Toronto Factor-Inwentash Faculty of Social Work, Canada

The authors should be commended for taking the reviewer feedback into consideration, which in turn substantially improved the manuscript. One reviewer you will see has minor revisions suggested that I agree with that will further strengthen the manuscript and make it closer to publication ready. We hope you can make these minor corrections.

# Editor report: Decision: Mindfulness, climate anxiety and psychological distress: a pathway to pro-environmental action in Pakistani emerging adults — R1/PR10

**DOI:** 10.1017/gmh.2026.10234.pr10

**Affiliations:** London School of Hygiene & Tropical Medicine, United Kingdom of Great Britain and Northern Ireland

No accompanying comment.

# Editor report: Recommendation: Mindfulness, climate anxiety and psychological distress: a pathway to pro-environmental action in Pakistani emerging adults — R2/PR12

**DOI:** 10.1017/gmh.2026.10234.pr12

**Affiliations:** University of Toronto Factor-Inwentash Faculty of Social Work, Canada

The authors have addressed the reviewer comments and the article is ready for publication. However small edits are needed:

-please place a 0 before the decimal place for all your calculations

-there is an extra 2 words (data screening) on line 276 that should be removed or put as a title (right now it is in the middle of a sentence)

-line 304: after you say interpretation caveat have a colon rather than a period

-in the discussion you talk about different theoretically plausible configurations in both paragraph 2 and 8-please consolidate in one place

Overall great work!

# Editor report: Decision: Mindfulness, climate anxiety and psychological distress: a pathway to pro-environmental action in Pakistani emerging adults — R2/PR13

**DOI:** 10.1017/gmh.2026.10234.pr13

**Affiliations:** London School of Hygiene & Tropical Medicine, United Kingdom of Great Britain and Northern Ireland

No accompanying comment.
